# Exercise and the Pulse-Rate

**Published:** 1910-06-25

**Authors:** 


The Hospital

A Journal of

The Medical Sciences and Hospital Administration.

New Series. No. 176, Vol. VII. [No. 1248, Vol. XLVIII.] SATURDAY, JUNE 25, 1910

### Exercise and the Pulse-Rate.

It is quite extraordinary what an amount of confusion and uncertainty exist over comparatively simple matters connected with pulse-rates. Whereas complicated and obscure problems such as those investigated in recent years by Dr. Mackenzie and many others have yielded gradually to the ingenuity and perseverance of researchers, on many points demanding a few easy observations no one seems to entertain more than vague and often inaccurate conceptions. For example, some medical authors would have readers believe that a pulse-rate of over 160 is uncountable; and many members of the profession look with scepticism upon anyone who counts a pulse-rate of 200 or 220 to the minute. Yet when one of these extremely rapid pulses is brought under observation it is quite easy to demonstrate that 240 is by no means an uncountable rate with no other appliances than an average finger and a watch with a seconds hand; indeed, after practice even this does not represent the limit of tactile perceptions. Again, Sir Clifford Allbutt in his "System of Medicine" draws the conclusion, from some experiments by Dr. James Kerr, that 172 is the extreme limit of pulse acceleration in the normal mechanism and that 160 is rarely attained. He believes that if the limit 170-172 be crossed there must be either some affection of the medulla or a shift of the cardiac rhythmical centre; and he has also committed himself to the opinion that for men in fair condition a doubled pulse-rate is excessive.

Now these and similar details with regard to the effect of exercise and exertion upon the rate of the heart-beat are capable very easily of test; and Lieut.-Colonel Deane, who has devoted considerable time to an investigation of them, has arrived at some very interesting conclusions which he has published in the *Journal of the Royal Army Medical Corps*. This officer's work was originally undertaken with the object of ascertaining how far the position of "attention" and the severe training course through which recruits are put might account for the "irritable heart" of soldiers; and it was in pursuing this line that he began to collect information by timing the pulses of soldiers and others after athletic sports and exercises. Professional gymnasts and ballet-dancers were also tested, as being people who must and do maintain themselves in a condition of physical fitness for severe daily exertion without the exclusive or modified dietary and unusual mode of life which are usually the lot of amateur athletes' "training" for a particular event. On one occasion the author tested men competing in teams for the Duke of Connaught's Cup at Aldershot, a competition which involves a race of over a mile with eleven difficult obstacles. He found that the pulse-rate just after the completion of the course was almost always double, sometimes nearly treble, its rate before starting. At an Army athletic meeting a number of observations were made upon a man, aged 24, who won a good many heats and events on the same afternoon. His pulse-rate was 76 to begin with; after running 100 yards it was 180, falling in successive quarter minutes to 164, 148, 156, 124. A quarter of an hour later he went in for the long jump, after which the rate was 136, dropping shortly to 120, at which it remained steady. Two hours later it was still 100, and the man then won the final of the 100 yards, after which the rate was 172. An hour and a half later he won a hurdle race, ending it with a pulse of 204. The next morning his pulse was steady at 60. Similar results were obtained from professional gymnasts and step-dancers, both male and female; and pulse-rates of 200 and upwards were noticed not infrequently even after only two minutes of energetic dancing. In fact, even with those who were giving turns in more than one theatre, and must therefore have been in the pink of condition, it seemed as if to double the pulse-rate is scarcely as much as is usually to be expected. The observations were numerous and uniform enough to exclude the possibility that the individuals tested were abnormal. In ballet-dancers the normal pulse-rate was usually regained within an hour or two after their performances. An even more interesting and inexplicable series of figures seems to show that in some cases a trained man's pulse takes longer to return to its normal rate than that of an untrained man who has undertaken the same feat. Lieut.-Colonel Deane points out, what is certainly true, that the untrained man does not really exert himself as much as the trained one, for the simple reason that he cannot; but even allowing for that source of error, he concludes that the statement that pulse acceleration is much more moderate and passes off sooner in trained than in untrained persons requires much qualification. Thus, he persuaded two people to run up and down 142 steps: one, trained, started with a pulse-rate of 63 and ended at 116; the other, untrained, started at 83 and finished at 160. The proportionate increases in the two cases were just about the same, and in both men it required half an hour for the pulse to return to within eight beats of the normal.

As for the bearings of these interesting facts upon the "irritable heart" of soldiers, the author concludes that the rapidity of the heart after exercise offers no guide. That is, that a great acceleration does not mean that the individual will develop that peculiar condition. Nor can any rules be laid down as to when the pulse should resume its normal rate after exertion. Yet another important point emerged during the discussion subsequent to the reading of Lieut.-Colonel Deane's paper; this is Dr. Mackenzie's statement that one of the most common forms of irregular action of the heart is not only not a sign of heart disease, but probably evidence that the heart is free from disease. This apparent paradox refers to that form of irregularity which has been called "sinus irregularity," where there is a constant variation in the duration of the diastolic period, usually due to and associated with the movements of respiration. In doubtful cases after a febrile illness Dr. Mackenzie regards this irregularity as evidence that the heart muscle has not been affected. It is quite clear that such an aid to prognosis will, if substantiated, be most valuable. Dr. Mackenzie seems to think his experience of this point not yet eno[illegible]gh to dogmatise on, but the impression he has formed is highly interesting.

